# P-895. Perceptions of Bias and Discrimination Among Antimicrobial Stewardship Providers

**DOI:** 10.1093/ofid/ofae631.1086

**Published:** 2025-01-29

**Authors:** Allison M Giuffre, Lindsay Taylor, Katie Cinnamon, Fauzia Osman, Jessica S Tischendorf

**Affiliations:** University of Wisconsin School of Medicine and Public Health, Department of Medicine, Madison, Wisconsin, Fitchburg, Wisconsin; University of Wisconsin School of Medicine and Public Health, Madison, Wisconsin; William S. Middleton Memorial Veterans Hospital, Madison, WI; University of Wisconsin School of Medicine and Public Health, Department of Medicine, Madison, Wisconsin, Fitchburg, Wisconsin; University of Wisconsin School of Medicine and Public Health, Madison, Wisconsin

## Abstract

**Background:**

Bias and discrimination influence the experience of many in healthcare, and antimicrobial stewardship providers are no exception as demonstrated by work documenting differential uptake of recommendations made by men and women stewards. In this mixed method study, we explored the perceptions and experiences of bias and discrimination among antimicrobial stewards.

**Figure:**
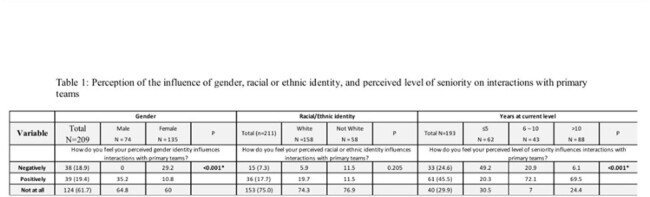

**Methods:**

We conducted a nationwide survey of stewardship providers including physicians, pharmacists, advanced practice providers and trainees. Participants were recruited via convenience sampling using X and professional listservs during May and June 2023. We solicited steward and program demographics and responses to statements exploring bias and discrimination in the execution of stewardship duties through a 67-item electronic survey (Qualtrics). Statistical analysis using chi-squared were performed in STATA. We further explored these experiences through semi-structured interviews.

**Figure:**
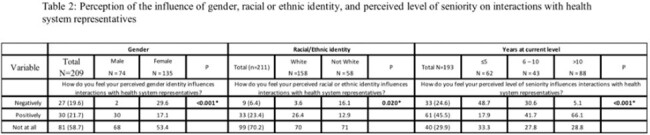

**Results:**

Stewards identifying as females and those with < =5 years were more likely to report their identities negatively influenced interactions with primary teams than male and more experienced stewards, respectively (table 1). Similarly, female stewards and those with the least experience reported these identities negatively influenced their interactions with health system representatives compared to male and more experienced stewards, respectively (table 2). Although there was no difference in the reported negative influence of race/ethnicity on interactions with primary teams, more non-white stewards reported their race/ethnicity negatively influenced interactions with health system representatives (tables 1 & 2). Themes from our 16 interviews illuminated sources of perceived bias, the impact they had, and strategies to mitigate the influence of these biases.

**Conclusion:**

Bias and discrimination are felt disproportionately by women and junior antimicrobial stewardship providers and can lead to poor job satisfaction and lack of perceived effectiveness. Acknowledging these experiences and equipping stewards with strategies to mitigate the harmful personal, professional and programmatic effects of bias and discrimination should be a priority of institutions and professional societies.

**Disclosures:**

**Jessica S. Tischendorf, MD, MS**, Merck: Grant/Research Support

